# Pre-radiotherapy cachexia is associated with treatment discontinuation in patients with head and neck squamous cell carcinoma receiving definitive radiotherapy

**DOI:** 10.3389/fonc.2026.1937723

**Published:** 2026-09-14

**Authors:** Qianrong Guo, Yan Sun, Yue Ni, Wenting Li, Lixin Wang, Minlei Zhu, Mianhua Wu

**Affiliations:** 1 First Clinical Medical College, Nanjing University of Chinese Medicine, Nanjing, Jiangsu, China; 2 Changzhou Hospital of Traditional Chinese Medicine Affiliated to Nanjing University of Chinese Medicine, Changzhou, Jiangsu, China; 3 Jiangsu Province Hospital of Chinese Medicine Affiliated to Nanjing University of Chinese Medicine, Nanjing, Jiangsu, China

**Keywords:** cachexia, definitive radiotherapy, head and neck squamous cell carcinoma, treatment discontinuation, treatment tolerance

## Abstract

**Purpose:**

This study aimed to evaluate the association between pre-radiotherapy cachexia and treatment discontinuation, as well as other treatment-related outcomes, in patients with head and neck squamous cell carcinoma (HNSCC) receiving definitive radiotherapy.

**Methods:**

This single-center retrospective cohort study consecutively enrolled M0 HNSCC patients who received definitive radiotherapy between July 2023 and December 2025. Cachexia was defined using an operational approach based on core diagnostic variables available in the medical records, informed by the Asian Working Group for Cachexia (AWGC) 2023 criteria. The primary outcome was treatment discontinuation. Secondary outcomes included unplanned radiotherapy interruptions, treatment-related adverse events, timing of acute radiation toxicity, timing of acute myelosuppression, nutritional risk during radiotherapy, and blood transfusion requirements. Logistic regression analyses were performed to assess the association between cachexia and treatment discontinuation.

**Results:**

A total of 137 patients were included, of whom 72 (52.6%) were classified as having cachexia. Treatment discontinuation was significantly higher in the cachexia group compared to the non-cachexia group (25.0% vs. 4.6%; P<0.001), and the incidence of treatment-related adverse events was also higher (97.2% vs. 78.5%; P<0.001). The distribution of first documented acute radiation toxicity across fraction-based categories differed between groups (P = 0.037). No significant differences were observed in unplanned interruptions, timing of acute myelosuppression, nutritional risk, or transfusion requirements. In univariable logistic regression, cachexia was significantly associated with treatment discontinuation (OR = 6.89; 95% CI 1.92–24.67; P = 0.003). After adjustment for age, Eastern Cooperative Oncology Group (ECOG) performance status, and concurrent chemotherapy, cachexia remained significantly associated with treatment discontinuation (OR = 6.05; 95% CI, 1.63–22.49; P = 0.007). Sensitivity analysis using Firth’s penalized logistic regression yielded consistent results.

**Conclusions:**

In M0 HNSCC patients receiving definitive radiotherapy, pre-radiotherapy cachexia is significantly associated with treatment discontinuation and is accompanied by a higher incidence of treatment-related adverse events. Assessment of cachexia prior to radiotherapy may provide clinically relevant information on treatment tolerance and help identify patients at high risk of therapy discontinuation.

## Introduction

Head and neck cancer is the seventh most common malignancy worldwide, with an increasing societal and healthcare burden (1). The management of localized head and neck cancer typically involves surgery, radiotherapy, or a combination of treatment modalities, and a proportion of patients achieve favorable long-term disease control (2). Radiotherapy plays a central role in the treatment of head and neck cancer and may be delivered as either definitive or adjuvant therapy (3). However, failure to complete planned radiotherapy remains a significant clinical challenge, encompassing unplanned radiotherapy interruptions and, more severely, treatment discontinuation. Previous studies have demonstrated that radiotherapy interruptions are associated with impaired locoregional control and survival, with longer interruptions resulting in greater adverse effects on treatment outcomes (4–6). Treatment discontinuation represents a more severe disruption of definitive radiotherapy and may adversely affect tumor control and long-term outcomes. Reported causes of definitive radiotherapy discontinuation include treatment-related toxicities, disease progression, comorbid conditions, patient refusal, and social factors (7), with most cases related to toxicity, clinical deterioration, or infection (8). Therefore, identifying patients at high risk of treatment discontinuation during radiotherapy is of considerable clinical importance.

Treatment-related adverse events are common among patients receiving definitive radiotherapy for head and neck cancer (9) and may contribute to physical burden and psychological distress, thereby compromising treatment adherence (10, 11). Oral mucositis is one of the most frequent acute toxicities and its severity has been associated with lower body mass index (BMI) and hemoglobin levels (12, 13). Hypoalbuminemia is also reported to reduce radiotherapy tolerance (14). Collectively, these findings suggest that nutritional status may be an important and clinically relevant factor influencing treatment adherence, treatment-related toxicities, and treatment completion.

Within the context of nutritional status, cachexia is a disease-related form of malnutrition accompanied by systemic inflammation (15). It is characterized by ongoing skeletal muscle loss, weight loss, and functional impairment (16), and may ultimately contribute to organ dysfunction and mortality (17). Cachexia is highly prevalent among patients with head and neck cancer and has been associated with dysphagia and poor outcomes after free flap reconstruction (18–20). Some studies have suggested that weight loss in head and neck cancer patients is primarily due to reduced oral intake caused by local symptoms (21). However, unlike simple insufficient intake, cachexia is generally characterized by tumor-associated metabolic disturbances and inflammation, and anorexia alone cannot fully explain its occurrence (22). Previous studies have reported that pre-radiotherapy cachexia is associated with poorer treatment outcomes and reduced adherence (23, 24), and pre-radiotherapy nutritional interventions may help attenuate nutritional deterioration, reduce treatment-related toxicities, and improve quality of life (25).

Patients with head and neck squamous cell carcinoma (HNSCC) are particularly susceptible to cachexia due to impaired nutritional intake and tumor-associated inflammatory responses (18). Previous studies have linked nutritional disorders with radiotherapy outcomes and identified factors associated with premature discontinuation of definitive radiotherapy (7). However, whether pre-radiotherapy cachexia is associated with treatment discontinuation in patients with HNSCC remains unclear. In Asian populations, due to differences in body composition, existing cachexia diagnostic criteria may have limited sensitivity for detecting early nutritional depletion (26). To address this, the 2023 Asian Working Group for Cachexia (AWGC) proposed diagnostic criteria tailored for Asian populations. In this study, we applied an operational definition informed by the AWGC 2023 framework using core variables available in retrospective medical records. This study aimed to investigate the association between pre-radiotherapy cachexia and treatment discontinuation in patients with HNSCC receiving definitive radiotherapy, and to explore the clinical relevance of cachexia assessment in identifying patients with increased risk of treatment discontinuation and poor treatment tolerance.

## Materials and methods

### Study design and participants

This single-center retrospective cohort study was conducted in the Department of Radiotherapy at Jiangsu Province Hospital of Chinese Medicine Affiliated to Nanjing University of Chinese Medicine. The study protocol was approved by the Ethics Committee of Jiangsu Province Hospital of Chinese Medicine (Approval No.: 2026NL-043-02). As this study used de-identified retrospective clinical data, the requirement for informed consent was waived. All procedures were conducted in accordance with the principles of the Declaration of Helsinki.

A total of 209 patients with head and neck tumors hospitalized in the radiotherapy department between July 2023 and December 2025 were consecutively screened through the hospital’s electronic medical record system. Inclusion criteria were (1): pathologically confirmed HNSCC; (2) age ≥18 years; (3) first-time receipt of definitive radiotherapy to the primary tumor, defined as radiotherapy delivered as the primary curative treatment without prior radical surgical resection; and (4) absence of distant metastasis (M0) confirmed by imaging. Exclusion criteria were: (1) incomplete medical records or missing key information; and (2) presence of other active malignancies. After applying these criteria, 137 patients were included. Patients were excluded for the following reasons: prior radiotherapy (n=31; 22 completed at external institutions, 9 partially completed at the outpatient clinic of this hospital), palliative radiotherapy (n=7), no radiotherapy (n=16), or non-squamous histology (n=18). The patient selection flowchart is presented in **Figure 1**.

**Figure 1:**
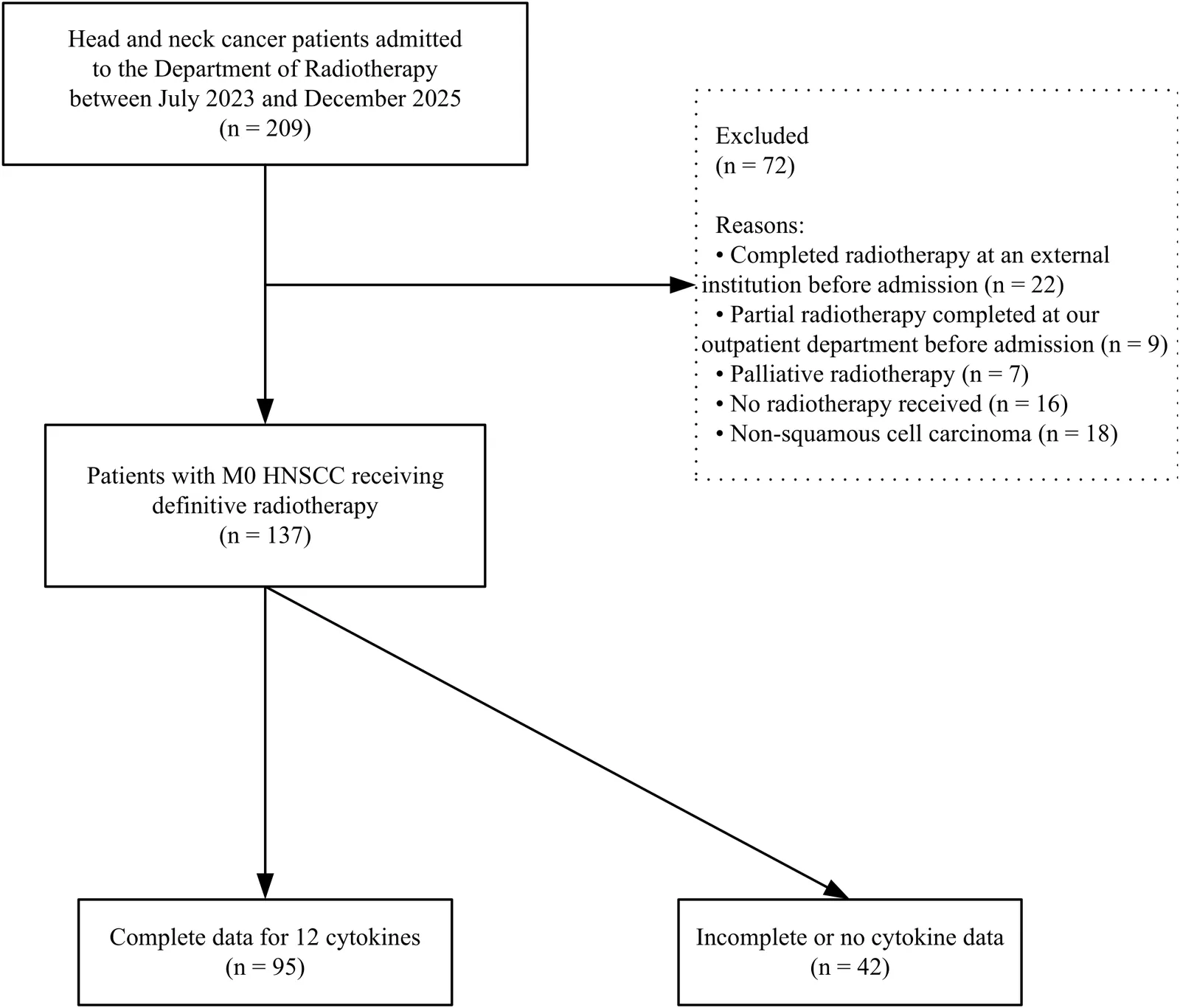
Flowchart of patient selection.

Among the 137 included patients, 95 had complete data for 12 cytokines. As cytokine testing was not routinely performed in all patients, this subset was used only for exploratory analyses. To assess representativeness, baseline characteristics were compared between patients with and without cytokine data.

### Data collection and quality control

All data were obtained from the hospital’s electronic medical record system, radiotherapy records, and laboratory information system. Clinical and treatment-related variables collected included sex, age, height, tumor site, planned radiotherapy dose, concurrent chemotherapy, comorbidities, smoking and alcohol status, marital status, occupation, Eastern Cooperative Oncology Group (ECOG) performance status, TNM stage, distance from the treatment center, and season of radiotherapy initiation. Concurrent chemotherapy regimens were recorded based on the actual systemic treatment administered during the radiotherapy course. Cachexia-related variables collected included body weight, BMI, involuntary weight loss within the previous 3–6 months, magnitude of weight loss, C-reactive protein (CRP), and anorexia. Weight loss was recorded in kilograms; patients without weight loss were coded as 0 kg. Laboratory parameters and cytokine data were additionally collected for exploratory analyses.

For statistical purposes, several variables were categorized as follows: sex (male/female); ECOG performance status (0 vs ≥1); concurrent chemotherapy (yes/no); smoking (never, current, former); alcohol consumption (never, current, former); tumor site (nasopharynx, oral cavity, larynx, hypopharynx, other); T stage (T1–T4); N stage (N0–N3); distance from the treatment center (>50 km defined as “far”); and season of radiotherapy initiation (spring, summer, autumn, winter). Planned radiotherapy dose was defined as the prescribed dose to the gross primary tumor target determined during the radiotherapy treatment planning stage before treatment initiation. Comorbidities included diabetes, hypertension, coronary heart disease, hypothyroidism, hepatic or renal dysfunction, COVID-19 infection during radiotherapy, and other chronic conditions explicitly documented in the medical record.

Selection bias was minimized through strict inclusion and exclusion criteria. Height and weight were measured by radiotherapy nurses following standardized procedures, and all laboratory tests were performed using the hospital’s uniform laboratory systems to reduce measurement bias. Radiotherapy planning followed departmental standard procedures, with all patients receiving CT simulation, image acquisition, target delineation, and intensity-modulated radiotherapy. All radiotherapy courses were delivered once daily, five fractions per week. The prescribed dose to the gross primary tumor target ranged from 69.76 to 70 Gy, delivered in 32–34 fractions with a fractional dose of 2.06–2.18 Gy. The prescribed dose to PGTVnd was 66–69.96 Gy in 32–33 fractions, while PCTV1 and PCTV2 received 60–60.06 Gy and 50.4–54.12 Gy, respectively. SIB was used in 35 patients (25.5%); the remaining patients received non-SIB schedules. Outcome measures were determined based on medical records, radiotherapy records, and laboratory data, with objective clinical definitions applied for treatment discontinuation, interruptions, and transfusion requirements to minimize information bias.

### Exposure and outcome measures

The primary exposure was pre-radiotherapy cachexia, assessed using a retrospective operationalization of the AWGC 2023 criteria based on variables available in the medical records. Consistent with the AWGC 2023 framework, patients were classified as having cachexia if they had either a BMI <21 kg/m² or involuntary weight loss >2% within the preceding 3–6 months, together with at least one additional criterion: documented anorexia/appetite loss or CRP >5 mg/L. Handgrip strength was not routinely available in the retrospective records and therefore could not be incorporated into the operational assessment. Previous studies indicate that such operational definitions based on available variables retain prognostic value (27, 28).

The primary outcome was treatment discontinuation, defined as failure to complete the planned definitive radiotherapy course. For patients who discontinued treatment, the primary reason for discontinuation, the number of radiotherapy fractions completed, and the cumulative dose delivered before discontinuation were further reviewed from the original medical and radiotherapy records. Reasons for discontinuation were categorized according to the principal clinical circumstance documented in the medical records. Secondary outcomes included unplanned radiotherapy interruptions, treatment-related adverse events, timing of acute radiation toxicity, timing of acute myelosuppression, nutritional risk during radiotherapy, and blood transfusion requirements. Unplanned radiotherapy interruptions were defined as unscheduled treatment pauses lasting at least one day, excluding scheduled breaks such as weekends or public holidays, followed by resumption and completion of the planned radiotherapy course at our institution. Treatment-related adverse events were retrospectively identified from medical records as clinically significant acute toxicities occurring during radiotherapy, including oral mucositis, pharyngeal mucositis, radiation dermatitis, and myelosuppression. Mild toxicities not requiring clinical management were not systematically documented. These events generally corresponded to Common Terminology Criteria for Adverse Events (CTCAE) version 5.0 grade ≥2 toxicity; however, specific grades were not retrospectively assigned because standardized CTCAE grading was not prospectively recorded. Timing of acute radiation toxicity and acute myelosuppression was categorized as no occurrence, <14 fractions, 14–30 fractions, or >30 fractions. These categories were used descriptively to indicate when the first event was documented relative to treatment progression and were not intended as validated clinical thresholds. Nutritional risk during radiotherapy was assessed using the Nutritional Risk Screening 2002 (NRS 2002); the presence of nutritional risk at any assessment during radiotherapy was considered positive. Blood transfusion requirement was defined as administration of packed red blood cells for anemia during radiotherapy, based on documented transfusion records.

### Statistical analysis

Continuous variables were first evaluated for normality using the Shapiro–Wilk test. Normally distributed variables were expressed as mean ± standard deviation and compared using independent-sample t-tests; non-normally distributed variables were expressed as median and interquartile range and compared using the Mann–Whitney U test. Categorical variables were expressed as counts and percentages and compared using the χ² test or Fisher’s exact test, as appropriate. Univariable and multivariable logistic regression analyses were performed to evaluate factors associated with treatment discontinuation. Considering the limited number of treatment discontinuation events, the multivariable model was restricted to the primary exposure (cachexia) and a limited number of clinically relevant covariates, including age, ECOG performance status, and concurrent chemotherapy, to reduce the risk of overfitting. Firth’s penalized logistic regression was additionally performed as a sensitivity analysis to assess the robustness of the findings in the setting of sparse data. An additional Firth sensitivity model further adjusted for hypopharyngeal tumor site to assess its potential confounding effect. A separate sensitivity analysis using Firth’s penalized logistic regression was performed after conservatively reclassifying patients whose cachexia classification depended on elevated CRP and who had either documented concurrent infection or an uncertain temporal relationship between infection and CRP measurement. Odds ratios (ORs) with 95% confidence intervals (95% CIs) were reported. For the exploratory cytokine analysis, P values were adjusted for multiple comparisons using the Benjamini–Hochberg false discovery rate (FDR) method. All tests were two-sided, and P<0.05 was considered statistically significant; for cytokine analyses, statistical significance was assessed using FDR-adjusted P values. Primary statistical analyses were conducted using IBM SPSS Statistics version 27.0, while Firth’s penalized logistic regression, Benjamini–Hochberg FDR adjustment, and visualization of logistic regression results were performed using R software (version 4.6.1).

## Results

### Baseline clinical characteristics

A total of 137 patients with M0 HNSCC receiving definitive radiotherapy were included in this study. Using an operational definition informed by the AWGC 2023 criteria and based on core variables available in retrospective medical records, 72 patients (52.6%) were classified as cachectic. Baseline clinical characteristics of the cachexia and non-cachexia groups are presented in **Table 1**. Except for age, no significant differences were observed between groups in sex, tumor site, planned radiotherapy dose, concurrent chemotherapy, comorbidities, TNM stage, ECOG performance status, smoking and alcohol status, marital status, occupation, distance from the treatment center, or season of radiotherapy initiation. The cachexia group was significantly older than the non-cachexia group (median 72.0 vs. 69.0 years; P = 0.016).

**Table 1:** Baseline clinical characteristics of patients with and without cachexia (*n* = 137).

Variable	Non-cachexia (*n* = 65)	Cachexia (*n* = 72)	*P* value
Sex, *n* (%)			0.991
Male	47 (72.3)	52 (72.2)	
Female	18 (27.7)	20 (27.8)	
Age, years, Median (IQR)	69.0 (54.5, 74.0)	72.0 (60.8, 79.0)	0.016
Height, cm, Median (IQR)	165.0 (160.0, 173.0)	166.5 (160.0, 170.0)	0.136
Tumor site, *n* (%)			0.149
Nasopharynx	18 (27.7)	12 (16.7)	
Oral cavity	7 (10.8)	5 (6.9)	
Larynx	5 (7.7)	5 (6.9)	
Hypopharynx	33 (50.8)	50 (69.4)	
Other sites	2 (3.1)	0 (0.0)	
Planned radiotherapy dose, Gy, Median (IQR)	70.00 (69.96, 70.00)	69.96 (69.96, 70.00)	0.489
Concurrent chemotherapy, *n* (%)	44 (67.7)	40 (55.6)	0.145
Comorbidities, *n* (%)			
Diabetes	7 (10.8)	6 (8.3)	0.627
Hypertension	23 (35.4)	24 (33.3)	0.801
Coronary heart disease	4 (6.2)	4 (5.6)	1.000
Hypothyroidism	1 (1.5)	4 (5.6)	0.369
Hepatic or renal dysfunction	3 (4.6)	2 (2.8)	0.668
COVID-19 infection	2 (3.1)	1 (1.4)	0.604
Other comorbidities	9 (13.8)	17 (23.6)	0.146
T stage, *n* (%)			0.298
T1	9 (13.8)	5 (6.9)	
T2	33 (50.8)	32 (44.4)	
T3	19 (29.2)	31 (43.1)	
T4	4 (6.2)	4 (5.6)	
N stage, *n* (%)			0.288
N0	25 (38.5)	29 (40.3)	
N1	23 (35.4)	22 (30.6)	
N2	9 (13.8)	17 (23.6)	
N3	8 (12.3)	4 (5.6)	
ECOG performance status, *n* (%)			0.351
0	8 (12.3)	13 (18.1)	
≥ 1	57 (87.7)	59 (81.9)	
Smoking status, *n* (%)			0.754
Never	43 (66.2)	50 (69.4)	
Current	7 (10.8)	9 (12.5)	
Former	15 (23.1)	13 (18.1)	
Alcohol consumption, *n* (%)			0.844
Never	45 (69.2)	53 (73.6)	
Current	8 (12.3)	8 (11.1)	
Former	12 (18.5)	11 (15.3)	
Marital status, *n* (%)			0.942
Unmarried	1 (1.5)	1 (1.4)	
Married	58 (89.2)	62 (86.1)	
Divorced	0 (0.0)	1 (1.4)	
Widowed	6 (9.2)	8 (11.1)	
Occupation, *n* (%)			0.155
Employee	11 (16.9)	8 (11.1)	
Retired	13 (20.0)	27 (37.5)	
Farmer	7 (10.8)	3 (4.2)	
Unemployed	13 (20.0)	12 (16.7)	
Other	21 (32.3)	22 (30.6)	
Distance from treatment center, *n* (%)			0.145
Near	21 (32.3)	32 (44.4)	
Far	44 (67.7)	40 (55.6)	
Season of treatment initiation, *n* (%)			0.204
Spring	27 (41.5)	19 (26.4)	
Summer	15 (23.1)	16 (22.2)	
Autumn	14 (21.5)	20 (27.8)	
Winter	9 (13.8)	17 (23.6)	

IQR, interquartile range. Continuous variables are presented as median (IQR); categorical variables are presented as n (%); ECOG, Eastern Cooperative Oncology Group; Far distance was defined as >50 km.

### Cachexia-related diagnostic indicators

Clinical parameters related to cachexia classification are summarized in **Table 2**. Compared with the non-cachexia group, patients with cachexia had significantly lower body weight and BMI (both P<0.001). The incidence of weight loss >2% within 3–6 months, CRP >5 mg/L, and anorexia were significantly higher in the cachexia group (all P<0.001), and the magnitude of weight loss was greater (P<0.001).

**Table 2:** Variables used in the operational cachexia assessment informed by the AWGC 2023 criteria (*n* = 137).

Variable	Non-cachexia (*n* = 65)	Cachexia (*n* = 72)	*P* value
Body weight, kg, Mean ± SD	67.22 ± 9.34	55.58 ± 8.03	<0.001
BMI, kg/m^2^, Median (IQR)	24.10 (22.72, 25.71)	20.66 (18.83, 22.23)	<0.001
Weight loss > 2% within 3–6 months, *n* (%)	4 (6.2)	34 (47.2)	<0.001
Magnitude of weight loss, kg, Median (IQR)	0.00 (0.00, 0.00)	0.50 (0.00, 3.00)	<0.001
CRP >5 mg/L, *n* (%)	4 (6.2)	27 (37.5)	<0.001
Anorexia, *n* (%)	9 (13.8)	57 (79.2)	<0.001

BMI, body mass index; IQR, interquartile range; SD, standard deviation. Continuous variables are presented as mean ± SD or median (IQR), as appropriate; categorical variables are presented as *n* (%). Patients without weight loss were recorded as 0 kg.

### Radiotherapy-related outcomes

Radiotherapy-related outcomes are summarized in **Table 3**. The rate of treatment discontinuation was significantly higher in the cachexia group compared with the non-cachexia group (25.0% vs. 4.6%; P<0.001). Among the 21 patients who discontinued radiotherapy, 16 (76.2%) discontinued in the context of documented treatment-related toxicity or poor treatment tolerance, including 15 patients with cachexia and 1 without cachexia. The remaining discontinuations were attributed to patient preference or refusal without a clearly documented treatment-related reason (n = 3, 14.3%), a social or family-related factor (n = 1, 4.8%), and an acute clinical event or intercurrent illness (n = 1, 4.8%). Patients who discontinued treatment completed a median of 22.0 radiotherapy fractions (IQR, 9.5–26.5), with a median cumulative delivered dose of 45.32 Gy (IQR, 20.44–55.37) (**Supplementary Table 1**). The incidence of treatment-related adverse events was also significantly elevated in cachectic patients (97.2% vs. 78.5%; P<0.001). The distribution of first documented acute radiation toxicity across fraction-based categories differed significantly between groups (P = 0.037). No significant differences were observed between groups in unplanned radiotherapy interruptions, timing of acute myelosuppression, nutritional risk during radiotherapy, or transfusion requirements.

**Table 3:** Treatment-related clinical outcomes according to cachexia status (*n* = 137).

Variable	Non-cachexia (*n* = 65)	Cachexia (*n* = 72)	*P* value
Treatment discontinuation, *n* (%)	3 (4.6)	18 (25.0)	<0.001
Unplanned radiotherapy interruption, *n* (%)	6 (9.2)	5 (6.9)	0.623
Adverse events, *n* (%)	51 (78.5)	70 (97.2)	<0.001
Acute radiation toxicity onset, *n* (%)			0.037
No occurrence	30 (46.2)	19 (26.4)	
<14 fractions	14 (21.5)	29 (40.3)	
14–30 fractions	20 (30.8)	22 (30.6)	
> 30 fractions	1 (1.5)	2 (2.8)	
Acute myelosuppression onset, *n* (%)			0.239
No occurrence	22 (33.8)	17 (23.6)	
<14 fractions	24 (36.9)	35 (48.6)	
14–30 fractions	19 (29.2)	18 (25.0)	
> 30 fractions	0 (0.0)	2 (2.8)	
Nutritional risk during radiotherapy, *n* (%)	31 (47.7)	42 (58.3)	0.213
Blood transfusion requirement, *n* (%)	1 (1.5)	3 (4.2)	0.621

Data are presented as *n* (%).

### Logistic regression analysis of treatment discontinuation

Factors associated with treatment discontinuation are summarized in **Table 4** and visually presented in **Figure 2**. In univariable analysis, cachexia was significantly associated with treatment discontinuation (OR = 6.89; 95% CI, 1.92–24.67; P = 0.003), and concurrent chemotherapy was associated with lower odds of treatment discontinuation (OR = 0.25; 95% CI, 0.10–0.68; P = 0.006). After adjustment for age, ECOG performance status, and concurrent chemotherapy, cachexia remained significantly associated with treatment discontinuation (OR = 6.05; 95% CI, 1.63–22.49; P = 0.007). Concurrent chemotherapy also remained associated with lower odds of treatment discontinuation (OR = 0.28; 95% CI, 0.10–0.77; P = 0.014), whereas age and ECOG performance status were not statistically significant.

**Table 4:** Univariable and multivariable logistic regression analyses for treatment discontinuation.

Variable	Univariable OR (95% CI)	*P* value	Multivariable OR (95% CI)	*P* value
Cachexia	6.89 (1.92–24.67)	0.003	6.05 (1.63–22.49)	0.007
Age, per year	1.02 (0.98–1.06)	0.297	1.01 (0.96–1.05)	0.812
ECOG performance status ≥1	0.73 (0.22–2.43)	0.608	0.71 (0.19–2.72)	0.618
Concurrent chemotherapy	0.25 (0.10–0.68)	0.006	0.28 (0.10–0.77)	0.014
Hypopharyngeal tumor site	1.36 (0.51–3.63)	0.536		
Distance from treatment center	0.65 (0.25–1.65)	0.363		

OR, odds ratio; CI, confidence interval; ECOG, Eastern Cooperative Oncology Group. Reference categories: non-cachexia, ECOG 0, no concurrent chemotherapy, non-hypopharyngeal site, and ≤50 km. Multivariable model adjusted for cachexia, age, ECOG performance status, and concurrent chemotherapy.

**Figure 2:**
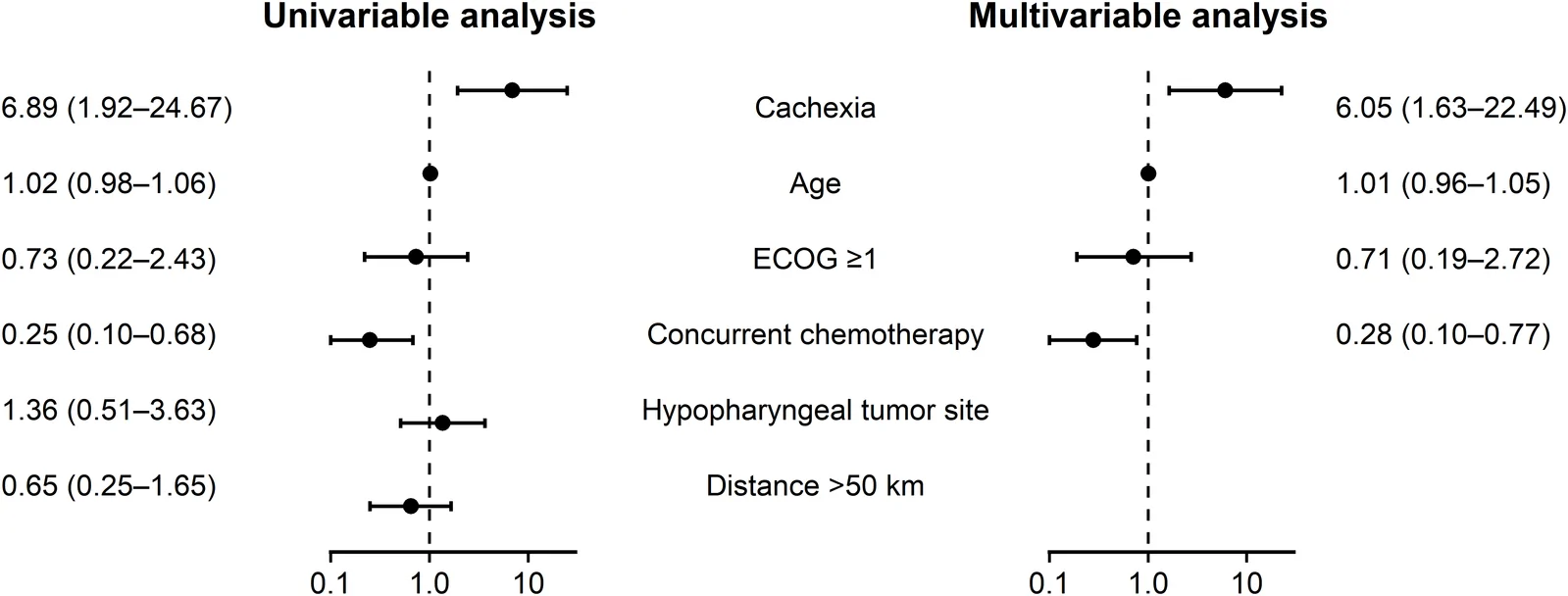
Forest plot of univariable and multivariable logistic regression analyses for treatment discontinuation.

Sensitivity analysis using Firth’s penalized logistic regression demonstrated similar results (**Supplementary Table 2**). Cachexia remained significantly associated with treatment discontinuation after adjustment for age, ECOG performance status, and concurrent chemotherapy (OR = 5.16; 95% CI, 1.67–20.79; P = 0.003). Further adjustment for hypopharyngeal tumor site did not materially change the association (OR = 5.01; 95% CI, 1.62–20.17; P = 0.004). The association also remained consistent after conservative reclassification addressing potential CRP-related misclassification in patients with documented concurrent infection or an uncertain temporal relationship between infection and CRP measurement (**Supplementary Table 3**).

### Exploratory analysis of nutritional and laboratory parameters

Exploratory analyses of laboratory and nutritional parameters are shown in **Table 5**. Cachectic patients had significantly lower serum albumin levels compared with non-cachectic patients (38.09 ± 4.11 vs. 40.44 ± 3.98 g/L; P<0.001), and prealbumin levels were also reduced (0.17 vs. 0.20 g/L; P = 0.001). In addition, the percentage of neutrophils was significantly higher in the cachexia group (P = 0.011). No significant differences were observed between groups in hemoglobin, white blood cell count, platelet count, or liver and kidney function indices.

**Table 5:** Exploratory nutritional and laboratory profiles according to cachexia status (*n* = 137).

Variable	Non-cachexia (*n* = 65)	Cachexia (*n* = 72)	*P* value
Alb, g/L, Mean ± SD	40.44 ± 3.98	38.09 ± 4.11	<0.001
PA, g/L, Median (IQR)	0.20 (0.16, 0.25)	0.17 (0.14, 0.22)	0.001
Hb, g/L, Median (IQR)	122.00 (108.50, 132.50)	117.00 (107.00, 126.75)	0.107
WBC, ×10^9^/L, Median (IQR)	5.15 (4.20, 6.20)	4.88 (3.96, 6.47)	0.826
PLT, ×10^9^/L, Median (IQR)	203.00 (154.00, 238.00)	194.00 (144.00, 265.25)	0.964
NEUT%, Median (IQR)	61.50 (53.95, 67.20)	65.65 (59.33, 72.40)	0.011
ALT, U/L, Median (IQR)	16.00 (11.50, 23.00)	13.50 (9.00, 21.75)	0.169
AST, U/L, Median (IQR)	18.00 (14.00, 24.00)	20.00 (15.00, 25.75)	0.279
Cr, μmol/L, Median (IQR)	69.00 (61.30, 78.75)	65.50 (55.25, 75.00)	0.080

IQR, interquartile range; SD, standard deviation; Alb, albumin; PA, prealbumin; Hb, hemoglobin; WBC, white blood cell count; PLT, platelet count; NEUT%, neutrophil percentage; ALT, alanine aminotransferase; AST, aspartate aminotransferase; Cr, creatinine. Continuous variables are presented as mean ± SD or median (IQR), as appropriate.

### Exploratory cytokine analysis

Among the 137 patients in the main cohort, 95 had complete cytokine data (non-cachexia, n=47; cachexia, n=48). Patients with cytokine data were generally similar to those without in most baseline characteristics, except for differences in N stage distribution (**Supplementary Table 4**). Results of exploratory cytokine analysis are presented in **Table 6**. In unadjusted analyses, IL-6 and IFN-γ levels were higher in cachectic patients than in non-cachectic patients (P = 0.048 and P = 0.032, respectively); however, neither remained statistically significant after FDR correction (both FDR-adjusted P = 0.212). No other cytokines showed statistically significant between-group differences after FDR correction.

**Table 6:** Exploratory cytokine analysis according to cachexia status (*n* = 95).

Cytokine, pg/mL, Median (IQR)	Non-cachexia (*n* = 47)	Cachexia (*n* = 48)	*Unadjusted P*	*FDR-adjusted P*
IL-5	3.55 (2.33, 4.85)	4.13 (2.39, 6.39)	0.326	0.559
IFN-α	2.29 (1.82, 2.93)	3.05 (1.94, 4.66)	0.080	0.219
IL-2	2.38 (1.75, 3.09)	2.77 (2.05, 3.89)	0.053	0.212
IL-6	5.96 (3.26, 10.21)	10.20 (3.79, 19.38)	0.048	0.212
IL-1β	6.67 (2.87, 13.55)	7.39 (3.77, 14.76)	0.463	0.641
IL-10	2.40 (2.04, 3.17)	2.84 (2.30, 3.41)	0.091	0.219
IFN-γ	8.79 (3.01, 13.26)	12.00 (5.55, 18.35)	0.032	0.212
IL-8	2.25 (0.38, 9.41)	4.70 (1.01, 14.39)	0.167	0.335
IL-17	5.77 (3.15, 8.71)	4.48 (2.91, 7.83)	0.696	0.759
IL-4	1.76 (1.34, 2.11)	1.75 (1.33, 2.04)	0.947	0.947
IL-12p70	1.73 (1.37, 1.98)	1.66 (1.45, 2.06)	0.517	0.641
TNF-α	2.12 (1.42, 3.26)	2.27 (1.50, 3.16)	0.534	0.641

IQR, interquartile range; IL, interleukin; IFN, interferon; TNF, tumor necrosis factor. Continuous variables are presented as median (IQR). P values were adjusted for multiple comparisons using the Benjamini–Hochberg FDR method.

## Discussion

In this study, we applied an operational definition based on core variables available in retrospective medical records, informed by the AWGC 2023 diagnostic framework, to assess pre-radiotherapy cachexia in patients with HNSCC. Our results demonstrated that cachectic patients had a significantly higher rate of treatment discontinuation compared with non-cachectic patients, and cachexia remained significantly associated with treatment discontinuation after adjustment for age, ECOG performance status, and concurrent chemotherapy. In addition, the cachexia group experienced a higher incidence of treatment-related adverse events and a different distribution of first documented acute radiation toxicity across fraction-based categories. These findings suggest that pre-radiotherapy cachexia may reflect patients’ overall tolerance to definitive radiotherapy and may help identify individuals at higher risk of treatment discontinuation.

The AWGC 2023 consensus provides a novel diagnostic framework for evaluating cachexia in Asian oncology patients. In a large multicenter study, Xie et al. (29) reported that cachexia defined according to the AWGC 2023 criteria was independently associated with poorer survival outcomes and higher disease burden. However, that study did not focus on head and neck cancer, nor did it examine completion of radiotherapy. Our study extends the application of the AWGC 2023 framework to patients with HNSCC receiving definitive radiotherapy, specifically focusing on treatment discontinuation, a clinically meaningful outcome. The prevalence of cachexia in our cohort was 52.6%, which may partly reflect the substantial nutritional vulnerability of patients with HNSCC, in whom tumor-related dysphagia and impaired oral intake are common. This relatively high prevalence should also be interpreted in the context of our retrospective operationalization of the AWGC 2023 framework based on available BMI, weight-loss, anorexia, and CRP data, rather than as a prevalence estimate derived from a complete prospective AWGC assessment. Compared with prior studies emphasizing weight loss, muscle mass reduction, or long-term survival, our results suggest that pre-radiotherapy cachexia may assist in identifying patients at higher risk of failing to complete curative radiotherapy.

During radiotherapy for head and neck cancer, patients frequently experience oral mucositis, dysphagia, reduced oral intake, and general deterioration, which may further exacerbate nutritional depletion and impact treatment completion (30). Further characterization of the discontinuation events showed that 16 of the 21 discontinuations occurred in the context of documented treatment-related toxicity or poor treatment tolerance, 15 of which occurred in patients with cachexia. This observation is consistent with the higher incidence of treatment-related adverse events in the cachexia group and provides additional clinical support for the interpretation that cachexia may reflect poorer treatment tolerance. However, some discontinuations were related to patient preference, social or family factors, or acute clinical events. Given the retrospective and observational nature of the study, these findings should not be interpreted as evidence that cachexia itself directly causes radiotherapy intolerance or treatment discontinuation. Notably, no significant difference was observed in unplanned radiotherapy interruptions between groups. Consistent with this, Choi et al. (31) reported no significant association between pre-radiotherapy weight loss and radiotherapy delays, and a meta-analysis by Schaeffers et al. (32) showed that low skeletal muscle mass was only marginally associated with radiotherapy interruptions. These findings collectively indicate that single nutritional or muscle-related metrics may not reliably predict temporary radiotherapy interruption but are more likely associated with whether treatment is ultimately completed. Unplanned interruptions may be influenced by short-term factors such as infection, temporary clinical scheduling, equipment availability, and social circumstances, which may explain why their association with pre-radiotherapy cachexia is less direct than that of treatment discontinuation.

Interestingly, in our study, nutritional risk during radiotherapy assessed by NRS 2002 did not differ between groups, whereas pre-radiotherapy cachexia effectively distinguished patients at risk of treatment discontinuation. This suggests that cachexia assessment and traditional nutritional screening tools may capture different dimensions of clinical risk. While NRS 2002 primarily identifies current nutritional risk, cachexia assessment integrates BMI, weight loss, anorexia, and inflammatory status, providing a more comprehensive evaluation of nutritional–metabolic vulnerability. Therefore, in patients with HNSCC undergoing definitive radiotherapy, cachexia assessment may provide complementary information beyond conventional nutritional screening and may help identify patients at high risk of treatment discontinuation.

Exploratory laboratory analyses showed that cachectic patients had significantly lower serum albumin and prealbumin levels than non-cachectic patients, whereas hemoglobin, white blood cell count, platelet count, and liver and renal function parameters did not differ significantly between groups. Albumin and prealbumin are commonly used indicators of nutritional reserves, and their reduction indicates that cachectic patients already exhibited marked nutritional depletion before radiotherapy. Previous studies have shown that hypoalbuminemia is associated with poorer radiotherapy tolerance and increased toxicity in head and neck cancer patients (14, 33). These findings suggest that pre-existing nutritional deficits in cachectic patients may contribute to impaired treatment tolerance. Since liver and renal function as well as major hematologic indices did not differ between groups, the association between cachexia and treatment discontinuation is unlikely to be primarily driven by baseline organ dysfunction or bone marrow reserve, but may instead indicate poorer baseline clinical vulnerability and treatment tolerance.

Cytokine analyses were performed in only 95 patients and were exploratory in nature. Although IL-6 and IFN-γ levels were higher in cachectic patients in unadjusted analyses, neither remained statistically significant after FDR correction. IL-6 and IFN-γ have been implicated in inflammatory processes associated with cancer cachexia (18, 34); however, the present findings should be considered hypothesis-generating rather than evidence of specific inflammatory mechanisms. In addition, the difference in N-stage distribution between patients with and without cytokine data may have introduced selection bias. Compared with cytokine markers, albumin and prealbumin demonstrated more consistent differences between groups, suggesting that nutritional depletion was more consistently reflected in the measured clinical parameters in this cohort. Das Neves et al. (35) reported that patients with head and neck cancer may continue to exhibit persistent cachexia following definitive chemoradiotherapy, highlighting that nutritional and metabolic issues may extend throughout the treatment course.

For other secondary outcomes, no significant associations were observed between cachexia and the timing of acute myelosuppression or transfusion requirements. The onset of myelosuppression may be influenced more by cumulative radiotherapy dose, concurrent chemotherapy regimen, and hematopoietic cell turnover rather than by pre-radiotherapy cachexia. Transfusion requirements were low in both groups, indicating that despite poorer nutritional reserves, cachectic patients in this cohort did not exhibit increased need for red blood cell transfusion. These negative findings help delineate the clinical impact of cachexia, which appears to be more prominently associated with treatment discontinuation and overall adverse events rather than all treatment-related outcomes.

This study has several notable clinical implications. First, the retrospective operationalization of the AWGC 2023 framework in patients with HNSCC undergoing definitive radiotherapy provides supplementary evidence for its potential clinical utility in Asian head and neck cancer populations. Second, by focusing on treatment discontinuation—a clinically meaningful endpoint that has received relatively limited attention in nutrition-related studies—our findings indicate that pre-radiotherapy cachexia may facilitate risk stratification before treatment. Third, the assessment parameters employed in this study are derived from routinely available clinical data, offering feasibility and practical applicability for early risk identification in clinical settings.

Several limitations should be acknowledged. First, this is a single-center retrospective study, which may be subject to selection bias, information bias, and residual confounding; the findings reflect associations rather than causality. Treatment-related toxicities were retrospectively identified from routine clinical documentation rather than prospectively graded using standardized CTCAE assessments, precluding reliable analysis by specific toxicity grade. Fraction-based timing analyses were descriptive and may have been affected by reduced opportunity for later-fraction events among patients who discontinued treatment early. In addition, the limited number of treatment discontinuation events resulted in relatively wide confidence intervals and reduced precision of the effect estimates, although Firth’s penalized logistic regression was performed as a sensitivity analysis to address potential sparse-data bias. Second, handgrip strength was not routinely available in the retrospective records; therefore, this component could not be incorporated into the operational assessment, which cannot be considered equivalent to a complete prospective AWGC 2023 assessment. Third, cytokine analyses were conducted in a subset of patients with a different N-stage distribution from those without cytokine data and reflect only a single pre-radiotherapy time point. Although FDR correction was applied for multiple comparisons, these exploratory findings require validation in larger, prospective cohorts. Future studies incorporating dynamic nutritional assessments and interventional strategies are warranted to determine whether early nutritional support or multimodal interventions can improve radiotherapy tolerance and completion rates.

## Conclusions

In M0 HNSCC patients receiving definitive radiotherapy, pre-radiotherapy cachexia was significantly associated with treatment discontinuation and was accompanied by a higher incidence of treatment-related adverse events. These findings indicate that pre-radiotherapy cachexia assessment may provide clinically relevant information on treatment tolerance and help identify patients at high risk of therapy discontinuation. Prospective, multicenter studies are needed to further validate the clinical utility of this assessment.

## Data Availability

The original contributions presented in the study are included in the article/**Supplementary Material**. Further inquiries can be directed to the corresponding author.
