# Supplementary material for: Pre-radiotherapy cachexia is associated with treatment discontinuation in patients with head and neck squamous cell carcinoma receiving definitive radiotherapy
Source: Front Oncol. 2026 Sep 14;16:1937723. doi: 10.3389/fonc.2026.1937723 (PMC13616658; doi:10.3389/fonc.2026.1937723)
Supplement: Supplementary file 1 [file Supplementaryfile1.docx]

## Supplementary Tables

**Table S1.** Summary of treatment discontinuation characteristics

| Characteristic | Overall  (*n* = 21) | Non-cachexia  (*n* = 3) | Cachexia  (*n* = 18) |
| --- | --- | --- | --- |
| Primary reason for treatment discontinuation, n (%) |  |  |  |
| Treatment-related toxicity or poor treatment tolerance | 16 (76.2) | 1 (33.3) | 15 (83.3) |
| Patient preference or refusal without a clearly documented treatment-related reason | 3 (14.3) | 1 (33.3) | 2 (11.1) |
| Social or family-related factor | 1 (4.8) | 1 (33.3) | 0 (0.0) |
| Acute clinical event or intercurrent illness | 1 (4.8) | 0 (0.0) | 1 (5.6) |
| Number of fractions completed, median (IQR) | 22.0 (9.5–26.5) | – | – |
| Cumulative dose delivered, Gy, median (IQR) | 45.32 (20.44–55.37) | – | – |

Abbreviations: *IQR*, interquartile range. Data are presented as *n* (%) or median (IQR), as appropriate.

**Table S2.** Sensitivity analyses using Firth’s penalized logistic regression for treatment discontinuation

| Variable | Firth model 1  OR (95% CI) | *P* value | Firth model 2  OR (95% CI) | *P* value |
| --- | --- | --- | --- | --- |
| Cachexia | 5.16 (1.67–20.79) | 0.003 | 5.01 (1.62–20.17) | 0.004 |
| Age, per year | 1.00 (0.97–1.05) | 0.846 | 1.00 (0.95–1.05) | 1.000 |
| ECOG performance status ≥1 | 0.68 (0.20–2.66) | 0.563 | 0.71 (0.20–2.78) | 0.596 |
| Concurrent chemotherapy | 0.30 (0.11–0.78) | 0.014 | 0.29 (0.10–0.78) | 0.014 |
| Hypopharyngeal tumor site |  |  | 1.17 (0.32–4.56) | 0.813 |

Abbreviations: *OR*, odds ratio; *CI*, confidence interval; *ECOG*, Eastern Cooperative Oncology Group. Model 1 included cachexia, age, ECOG performance status, and concurrent chemotherapy. Model 2 additionally adjusted for hypopharyngeal tumor site. ORs and 95% CIs were estimated using Firth’s bias-reduced penalized logistic regression. Reference categories were non-cachexia, ECOG 0, no concurrent chemotherapy, and non-hypopharyngeal site.

**Table S3**. Sensitivity analysis after conservative reclassification addressing potential infection-related CRP misclassification

| Variable | OR (95% CI) | *P* value |
| --- | --- | --- |
| Cachexia (reclassified definition) | 5.71 (1.86–22.91) | 0.002 |
| Age, per year | 1.00 (0.97–1.05) | 0.816 |
| ECOG performance status ≥1 | 0.67 (0.19–2.63) | 0.549 |
| Concurrent chemotherapy | 0.30 (0.11–0.79) | 0.015 |

Abbreviations: *OR*, odds ratio; *CI*, confidence interval; *ECOG*, Eastern Cooperative Oncology Group. Three CRP-dependent cachexia cases with documented or temporally uncertain infection were conservatively reclassified as non-cachectic. Estimates were derived from Firth’s penalized logistic regression.

**Table S4**. Comparison of baseline characteristics between patients with and without available cytokine data

| Variable | Without cytokine data (*n* = 42) | With cytokine data (*n* = 95) | *P* value |
| --- | --- | --- | --- |
| Sex, *n* (%) |  |  | 0.072 |
| Male | 26 (61.9) | 73 (76.8) |  |
| Female | 16 (38.1) | 22 (23.2) |  |
| Age, years, Median (IQR) | 72.5 (66.5, 77.3) | 69.0 (57.0, 76.0) | 0.123 |
| Tumor site, *n* (%) |  |  | 0.668 |
| Nasopharynx | 8 (19.0) | 22 (23.2) |  |
| Oral cavity | 2 (4.8) | 10 (10.5) |  |
| Larynx | 3 (7.1) | 7 (7.4) |  |
| Hypopharynx | 29 (69.0) | 54 (56.8) |  |
| Other sites | 0 (0.0) | 2 (2.1) |  |
| Planned radiotherapy dose, Gy, Median (IQR) | 70.00 (69.96, 70.00) | 70.00 (69.96, 70.00) | 0.990 |
| Concurrent chemotherapy, *n* (%) | 28 (66.7) | 56 (58.9) | 0.392 |
| T stage, *n* (%) |  |  | 0.748 |
| T1 | 4 (9.5) | 10 (10.5) |  |
| T2 | 20 (47.6) | 45 (47.4) |  |
| T3 | 17 (40.5) | 33 (34.7) |  |
| T4 | 1 (2.4) | 7 (7.4) |  |
| N stage, *n* (%) |  |  | 0.003 |
| N0 | 24 (57.1) | 30 (31.6) |  |
| N1 | 7 (16.7) | 38 (40.0) |  |
| N2 | 10 (23.8) | 16 (16.8) |  |
| N3 | 1 (2.4) | 11 (11.6) |  |
| ECOG performance status, *n* (%) |  |  | 0.210 |
| 0 | 4 (9.5) | 17 (17.9) |  |
| ≥ 1 | 38 (90.5) | 78 (82.1) |  |
| Distance from treatment center, *n* (%) |  |  | 0.217 |
| Near | 13 (31.0) | 40 (42.1) |  |
| Far | 29 (69.0) | 55 (57.9) |  |
| Cachexia | 24 (57.1) | 48 (50.5) | 0.475 |
| Treatment discontinuation | 8 (19.0) | 13 (13.7) | 0.422 |

Abbreviations: *IQR*, interquartile range. Continuous variables are presented as median (IQR); categorical variables are presented as *n* (%); *ECOG*, Eastern Cooperative Oncology Group.
